# Bronchiectasis in severe asthma is associated with eosinophilic airway inflammation and activation

**DOI:** 10.1016/j.jacig.2022.10.001

**Published:** 2022-11-21

**Authors:** Laurits Frøssing, Anna Von Bülow, Celeste Porsbjerg

**Affiliations:** a Respiratory Research Unit, Department of Respiratory Medicine, Bispebjerg and Frederiksberg Hospital, Copenhagen, Denmark; b Copenhagen Center for Translational Research, Bispebjerg and Frederiksberg Hospital, Copenhagen, Denmark

**Keywords:** Severe asthma, bronchiectasis, airway inflammation, eosinophil activation, eosinophil degranulation, eosinophils, messenger RNA, free eosinophil granules

## Abstract

**Background:**

Bronchiectasis is a common comorbidity in severe asthma; causative pathogenic mechanisms are not fully understood but may differ from other causes of bronchiectasis. The role of eosinophilic airway inflammation, a classic feature of asthma predominantly driven by IL-5 and IL-13, in bronchiectasis is unclear, but association with disruption of the airway epithelium through eosinophil degranulation and increased mucus production is plausible.

**Objective:**

We sought to describe the prevalence of bronchiectasis in an unselected population of patients with severe asthma, and the association with the airway eosinophilic inflammation and activation.

**Methods:**

All patients with severe asthma according to European Respiratory Society/American Thoracic Society criteria (high-dose inhaled corticosteroids/oral corticosteroids), attending 4 respiratory clinics over a 1-year period, were included. All patients underwent high-resolution computed tomography and induced sputum was collected and analyzed for a cell differential count, free eosinophilic granules, and airway messenger RNA expression of T2 inflammatory pathways.

**Results:**

Bronchiectasis was present in 31% (34 of 108) of patients with severe asthma, and half (52%) of these patients had airway eosinophilia whereas only 16% of patients without bronchiectasis had airway eosinophilia. Patients with bronchiectasis had a significantly higher sputum eosinophil count (5.3 vs 0.8; *P* = .001) as well as more extensive eosinophil degranulation, compared with those without bronchiectasis (13% vs 2%; *P* = .05), suggesting a higher degree of eosinophil activation. Pairwise analyses identified significantly higher messenger RNA expression of *Charcot-Leyden crystal galectin* in patients with bronchiectasis (*P* = .02).

**Conclusions:**

Bronchiectasis in severe asthma was associated with eosinophilic airway inflammation and eosinophilic degranulation as well as messenger RNA expression of *Charcot-Leyden crystal galectin*.

Bronchiectasis is a common comorbidity in severe asthma, affecting 25% to 67% of patients, in whom it is associated with an increased risk of exacerbations.^1^, ^2^, ^3^ Severe asthma is increasingly recognized as a heterogeneous entity with different clinical, inflammatory, and molecular phenotypes and although bronchiectasis historically has been linked to neutrophilic airway inflammation, more recent evidence points to a subset with eosinophilic airway inflammation.^4^, ^5^, ^6^, ^7^

At present, our understanding of the inflammatory mechanisms resulting in eosinophil bronchiectasis and the association between eosinophilia in severe asthma and the development of bronchiectasis is still to be uncovered. A recent study from the Severe Asthma Research Programme severe asthma population found that bronchiectasis in severe asthma is associated with repetitive mucus plugging of the same bronchial segment.^8^ Mucus plugging was associated with eosinophilia and appeared to be promoted by release of eosinophil peroxidase (EPO). Hence, in severe asthma, eosinophilic activation with release of cytotoxic mediators causing oxidative stress could result in the repeated formation of sticky mucus plugs, and ultimately in damage to the airway wall and development of bronchiectasis. The most evident association between eosinophils and bronchiectasis in severe asthma is seen in patients who develop allergic bronchopulmonary aspergillosis (ABPA): As a result of interaction with *Aspergillus fumigatus*, eosinophils undergo extracellular trap cell death, and release large amounts of cytoxic proteins, thereby inducing a vicious circle of sticky mucus, decreased airway clearance of fungi, tissue damage, and formation of bronchiectasis.^9^

Eosinophilic airway inflammation is driven by T_H_2 cells and type 2 innate lymphoid cells, with IL-4, IL-13, and IL-5 considered the key signaling pathways. IL-4 signaling is central to B-cell class switching and triggers the release of IgE from B cells.^10^ IL-5 is critical for the differentiation, maturation, and recruitment of eosinophils as well their activation,^11^ whereas IL-13 signaling leads to the differentiation of bronchial epithelial cells into mucus-producing goblet cells.^12^

We hypothesized that bronchiectasis in severe asthma is caused by disruption of the airway epithelium, relating to active eosinophilic airway inflammation with degranulation of toxic proteins, as well as increased mucus production, relating to the transition of the epithelium to mucus cell hyperplasia caused by IL-5– and IL-13–driven pathways, respectively.

Therefore, we have in this study examined the relationship between the presence of bronchiectasis in severe asthma, the level of eosinophilic airway inflammation and degranulation, as well as activation of inflammatory pathways relating to IL-5 and IL-4/13.

## Methods

### Study design

As previously published,^13^ patients with severe asthma were recruited from 4 respiratory outpatient clinics in Denmark in a cross-sectional design with the aim of selecting a clinically representative population.

All patients with a diagnosis of asthma (*International Classification of Diseases, Tenth revision* codes DJ45.0-DJ45.9) seen in the respiratory clinics over 12 months (beginning October 2015) were screened to identify those currently receiving high-dose asthma treatment.^14^

Criteria for study inclusion were a physician’s diagnosis of asthma and high-dose inhaled corticosteroid treatment (≥1600 μg budesonide or equivalent) with a second controller (long acting β-agonist, theophylline, or leukotriene-antagonist) for the previous year or oral corticosteroids for 50% or more of the previous year.^14^ Exclusion criteria were as follows: age below 18 years, pregnancy, or judged by the investigator to be unable to comply with the study protocol.

### Assessments

All included patients were referred for a high-resolution computed tomography of thorax as per the study protocol, and patients were classified as having bronchiectasis if their high-resolution computed tomography was described with bronchiectasis by a thorax radiologist.

Radiologic severity of bronchiectasis was classified as outlined in the Bronchiectasis Severity Index as involvement of fewer than 3 lobes, involvement of 3 or more lobes, and cystic bronchiectasis.^15^

A MasterScreenPneumo spirometer and MasterScreenBodyBox (BD Jaeger, Würzburg, Germany) were used for spirometry and bodybox, respectively. Predicted values were calculated using the National Health and Nutrition Examination Survey reference data.^16^ Fractional exhaled nitric oxide was measured using the Ecomedics CLD88sp (Ecomedics AG, Duernten, Switzerland).

Skin prick test and specific serum IgEs (radioallergosorbent test) were performed using a standard panel including pollen from birch (*Betula verrucosa*), grass (*Phleum pratense*), ragweed (*Ambrosia psilostachya*), and mugwort (*Artemisia vulgaris*); dander from horse (*Equus caballus*), cat (*Felis domesticus*), and dog (*Canis familaris*); house-dust mites: *Dermatophagoides pteronyssinus* and *Dermatophagoides farina*; and the fungi: *Alternaria alternata/tenuis*, *A fumigatus*, and *Cladosporium herbarum*.

Atopy was defined as a positive skin prick test (≥3 mm) result or an elevated specific IgE level (>0.35 kU/L) for at least 1 of 12 tested aeroallergens.

### Sputum

Sputum was induced using incremental doses of hypertonic saline or isotonic saline if FEV was less than 70% predicted. The sputum sample was processed using the plug selection method as described by Bafadhel et al.^17^^,^^18^ A minimum of 2 plugs were selected: 1 for cell differential count (CDC) and 1 for gene expression analysis. When restricted by sample quantity, the CDC was prioritized. Sputum was not assessed for bacterial colonization.

Plugs for gene analyses were stored in RNAlater and frozen at −80°C. Gene analyses were performed at Hunter Medical Research Institute, Newcastle, Australia.

For the CDC, cutoff values for eosinophilia and neutrophilia in sputum were greater than or equal to 3% and greater than or equal to 61%, respectively.^18^

### Considerations regarding ABPA

Patients with a total IgE level of greater than or equal to 1000 IU/mL and either a positive skin prick test result or a positive radioallergosorbent test result for *A fumigatus* were considered to be suspicious of having ABPA in line with the criteria outlined by the *ABPA Complicating Asthma ISHAM Working Group*.^19^ To accommodate the emerging support for a lowering of the IgE threshold in the diagnosis of ABPA, a secondary analysis using a cutoff value of IgE level greater than or equal to 500 IU/mL was also performed.^20^^,^^21^

### Measurements of airway eosinophil activity

As a surrogate marker for eosinophil activation and degranulation, sputum was assessed (unblinded) for free eosinophil granules (FEGs).^22^, ^23^, ^24^ The amount of FEGs in cytospin sputum preparations was ranked by a trained lab technician on a semi-quantitative scale anchored from 0 to 3, representing a range (FEG score) from 0 = “none,” 1 = “few,” 2 = “moderate,” to 3 = “extensive.” When used as a dichotomous variable, FEG scores of 0 to 1 are termed “no degranulation” and FEG scores of 2 to 3 are termed “degranulation.”

### Gene expression analyses

As published previously, genes related to IL-5 (Charcot-Leyden crystal galectin [*CLC*], carboxypeptidase A3 [*CPA3*], and deoxyribonuclease gamma 1 like 3 [*DNASE1L3*]), IL-13 (IL-13 receptor subunit alpha-1 [*IL13Ra1*], tumor necrosis factor superfamily member 14 [*TNFSF14]*, and Serpin family B member 2 [*SERPINB2*]), T1/T_H_17 activity (IL-1β, alkaline phosphatase, tissue-nonspecific isozyme [*ALPL*], and C motif chemokine receptor 2 [*CXCR2*]), and *in vitro* response to corticosteroids (FKBP prolyl isomerase 2 [FKBP512]) and mepolizumab (ArfGAP With RhoGAP Domain, Ankyrin Repeat And PH Domain 3 [*ARAP3*]) were analyzed. Two housekeeping genes were measured (*18s* and *beta-actin*), and the difference in cycle threshold (ΔCt) for each target gene was calculated relative to 18s because it had the most consistent expression. Samples were considered of inadequate quality if expression of 18s was below 22 cycles.

The fold change in cycle threshold (2^−ΔCt^) between the target gene and the housekeeping gene (18s) and the fold abundance in expression relative to the mean expression in a previously published healthy control population were calculated (2^−ΔΔCt^).^5^^,^^25^

### Statistical analysis

SAS Enterprise guide version 7.1 (SAS Institute, Cary, NC) was used for data analyses.

Parametric and nonparametric continuous variables are presented as mean and median (quartile-1 and quartile-3) and were tested using Welch ANOVA or Kruskal-Wallis, respectively. Categorical variables were tested using χ^2^ or Fisher exact test as appropriate. In the exploratory subgroup analyses, a *P* value of .0025 was considered significant to correct for multiple comparisons.

Association between gene expression (2^−ΔCT^) and bronchiectasis was evaluated using multiple logistic regression gene expression and simple comparison of means.

To accommodate for the potential interference of ABPA, significant statistical tests were rerun in patients without suspicion of ABPA as outlined above.

### Ethics

The SATS (H-12014047) was approved by the local Scientific Ethics Committee, and written informed consent was signed by all participants.

## Results

**Fig 1:**
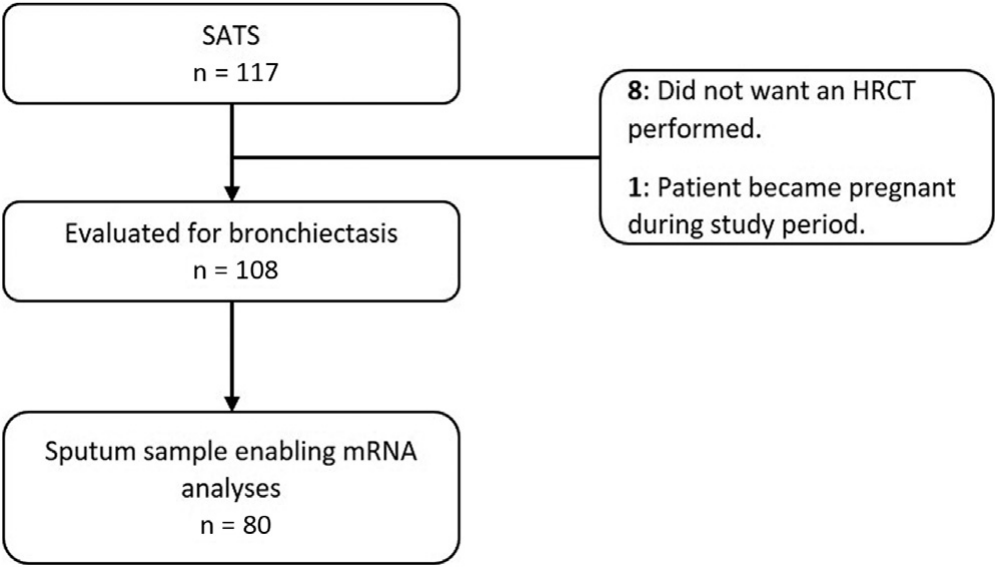
Flowchart visualizing the number of patients available for analyses of bronchiectasis and airway inflammation. *HRCT*, High-resolution computed tomography; *mRNA*, messenger RNA.

For this study, we included 108 of the 117 patients originally included in the SATS study because 9 were excluded for not having a high-resolution computed tomography performed (Fig 1). Of these, 79% (85 of 108) had a sputum sample enabling a CDC and 74% (80 of 108) a sample enabling messenger RNA analyses.

Bronchiectasis (BE) was present in 34 (31%; 34 of 108) patients with severe asthma (Fig 1, *A*); 29% (10 of 34) had involvement of 3 or more lobes; 71% (24 of 34) had involvement of fewer than 3 lobes, and none had cystic bronchiectasis. Baseline characteristics and inflammatory markers are presented in Table I and Table II, respectively. Patients with bronchiectasis were significantly older than patients without (52 vs 45 years; *P* = .01). We found no difference in smoking status or pack years between the 2 groups.

**Table I:** Baseline characteristics

Characteristic	All	Bronchiectasis	Nonbronchiectasis	*P*
Patients total, n	108	34	74	
Age (y)	47 ± 14.3	52 ± 14	45 ± 14	.01∗
Sex: female	63 (58)	18 (53)	45 (61)	.53†
BMI (kg/m^2^)	28 ± 6	27 ± 5	29 ± 6	.13‡
ACQ-5 score	1.7 (1.0-2.6)	1.7 (0.8-2.6)	1.7 (1.0-2.6)	.43‡
Cough and phlegm (preceding 12 mo)	85 (79)	30 (88)	55 (74)	.06†
Cough and phlegm for 3 consecutive months (preceding 2 y)	49 (45)	20 (59)	29 (39)	.05†
Courses of antibiotics (preceding 12 mo)	1 (0-2)	1 (0-3)	1 (0-2)	.14†
Courses of OCSs (preceding 12 mo)	1 (0-3)	1 (0-2)	1 (0-3)	.77‡
Never smokers	63 (58)	17 (50)	46 (62)	.29†
Smoking (pack years)	9 (3-21)	9 (7-35)	10 (3-20)	.34‡
Atopy	56 (52)	21 (62)	45 (61)	.21§
Aspergillus positive||	11 (10)	8 (24)	3 (4)	.004§
Aspergillus positive||*and* IgE > 1000 IU/mL	3 (3)	2 (6)	1 (1)	.23§
Cumulative ICS dose	1800 (1600-2400)	1600 (1600-2400)	2200 (1600-2400)	.19‡
OCSs for asthma	15 (14)	6 (18)	9 (12)	.55§
**Lung function**				
FEV_1_ % predicted	75 (21)	72 (25)	77 (19)	.37∗
FVC % predicted	87 (17)	86 (18)	87 (16)	.85∗

Data are presented as n (%), mean ± SD, or median (IQR).

*BMI*, Body mass index; *FVC*, forced vital capacity; *ICS*, inhaled corticosteroid; *IQR*, interquartile range; *OCS*, oral corticosteroid.

∗ Welch ANOVA.

† χ^2^ test.

‡ Kruskal-Wallis test.

§ Fisher exact test.

|| Aspergillus positive: positive skin prick test result and/or specific IgE for *A fumigatus*.

**Table II:** Inflammatory markers

Inflammatory marker	All	Bronchiectasis	Nonbronchiectasis	*P*
**Biomarkers (n = 108)**				
Blood eosinophils (cells × 10^9^/L)	0.20 (0.11-0.36)	0.24 (0.12-0.51)	0.19 (0.1-0.3)	.09∗
Sputum eosinophils (%)	1.5 (0.3-6.5)	5.3 (1.5-38)	0.8 (0.1-2.5)	.001∗
Sputum neutrophils (%)	53 (24-77)	54 (34-75)	52 (23-78)	.71∗
IgE total (IU/mL)	100 (19-229)	160 (62-436)	70 (16-207)	.006∗
Feno (ppb)	21 (12-33)	21 (12-30)	20 (11-34)	.75∗
**Eosinophil activity (n = 74)**				
Degranulation (FEG 2-3)	18 (24)	8 (35)	10 (20)	.16†
FEGs 0	46 (62)	12 (52)	34 (67)	.23†
FEGs 1	10 (14)	3 (13)	7 (14)	.93†
FEGs 2	14 (19)	5 (22)	9 (18)	.67†
FEGs 3	4 (5)	3 (13)	1 (2)	.05‡

Data are presented as n (%) or median (IQR).

*FEG*, Free eosinophil granule; *F**eno*, fractional exhaled nitric oxide; *IQR*, interquartile range; *ppb*, parts per billion.

∗ Kruskal-Wallis test.

† χ^2^ test.

‡ Fisher exact test.

Chronic bronchitis (cough and phlegm for 3 consecutive months in the preceding 2 years) was significantly more prevalent in patients with bronchiectasis (59% vs 39%; *P* = .05), whereas symptoms of bronchitis (cough and phlegm in the preceding 12 months) trended toward a higher prevalence only in patients with bronchiectasis (88% vs 74%; *P* = .06). There was no difference in the number of asthma exacerbations or courses of antibiotics.

Total IgE level was significantly higher in patients with BE (160 vs 90 IU/mL; *P* = .006), but we observed no difference in allergic sensitization (62% vs 61%; *P* = .2) nor in the level of fractional exhaled nitric oxide (21 parts per billion vs 20 parts per billion; *P* = .8) in patients with and without bronchiectasis.

Overall, 11 (10%; 11 of 108) patients were sensitized to *A fumigatus*; however, a positive aspergillus test result and total IgE level of more than 500 and more than 1000 IU/mL were present in only 3 (3%; 3 of 108) and 4 (4%, 4 of 108) patients, respectively. A significantly higher proportion of patients with bronchiectasis was sensitized to *A fumigatus* (24% vs 4%; *P* = .004); however, no difference in the prevalence of patients with concomitant aspergillus sensitization and IgE elevation (IgE > 500: 10% vs 1%, *P* = .08 and IgE > 1000: 6% vs 1%, *P* = .2) was identified.

**Fig 2:**
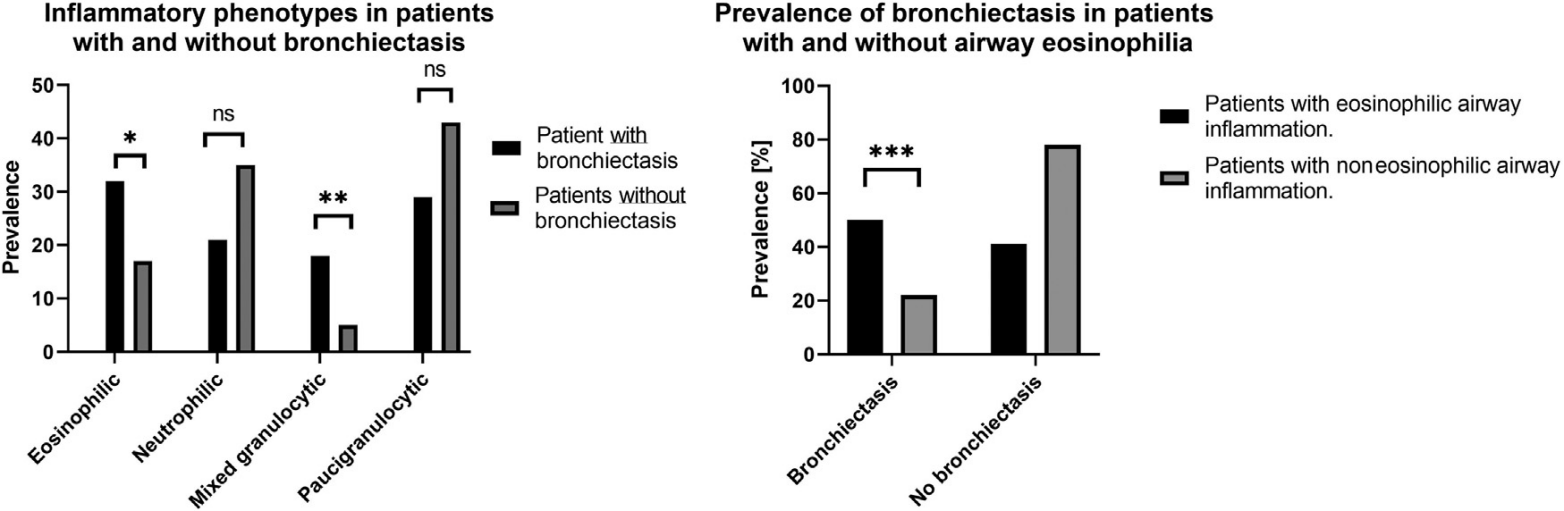
Associations between bronchiectasis and inflammatory phenotype in sputum: Eosinophilic (eosinophils ≥3% and neutrophils <61%), neutrophilic (eosinophils <3% and neutrophils ≥61%), mixed granulocytic (eosinophils ≥3% and neutrophils ≥61%), and paucigranulocytic (eosinophils <3% and neutrophils <61%). *ns*, Nonsignificant. Significance levels: ∗*P* = .0002 (χ^2^), ∗∗*P* = .02 (Fisher exact test), ∗∗∗*P* = .02 (χ^2^).

As presented in Fig 2, eosinophilic airway inflammation was significantly more prevalent in patients with bronchiectasis (50% vs 16%; *P* = .02), where half of the patients had airway eosinophilia (eosinophilic: 32% and mixed granulocytic: 18%), whereas only 16% of patients without bronchiectasis had airway eosinophilia (eosinophilic: 12% and mixed granulocytic: 4%). Unsurprisingly, the magnitude of eosinophil inflammation was significantly larger in patients (n = 10) with purely eosinophilic airway inflammation (eosinophils: 56.8% [38-64] and neutrophils: 28.8% [9.5-49.5]) compared with those with mixed granulocytic inflammation (n = 6) (eosinophils 6.4% [5.5-6.5] and neutrophils 86.1% [74.3-88.3]).

Sputum eosinophil count was significantly higher in patients with bronchiectasis compared with those without (5.3% vs 0.8%; *P* = .001). This remained statistically significant in patients without (n = 97) sensitization to *A fumigatus* (*P* = .005). The prevalence of “extensive” (FEGs 3) eosinophil degranulation was significantly higher in patients with bronchiectasis.

We found no difference in lung function in patients with and without bronchiectasis, but patients with concomitant bronchiectasis and sputum eosinophilia had a significantly lower FEV_1_ (58% vs 70%; *P* = .004) and lower functional vital capacity (79% vs 84%; *P* = .05) and were more obstructive (FEV_1_/forced vital capacity) (0.57 vs 0.66; *P* = .004) compared with those with bronchiectasis without sputum eosinophilia.

Patients with bronchiectasis involving 3 or more lobes had a significantly higher blood eosinophil count (0.62 vs 0.18 × 10^9^/L; *P* = .03) and total IgE (783 vs 127 IU/mL; *P* = .02) but not sputum eosinophil count (21% vs 4.3%; *P* = .2) compared with those involving fewer than 3 lobes. This remained significant for blood eosinophil count (0.80 vs 0.16 × 10^9^/L; *P* = .01) but not total IgE (512 vs 129 IU/mL; *P* = .3) in patients without sensitization to *A fumigatus*.

No significant associations between degranulation and radiologic severity of BE were observed despite absence of degranulation being more prevalent in patients with involvement of fewer than 3 lobes (62% vs 14%; *P* = .07) compared with those with involvement of 3 or more lobes.

**Fig 3:**
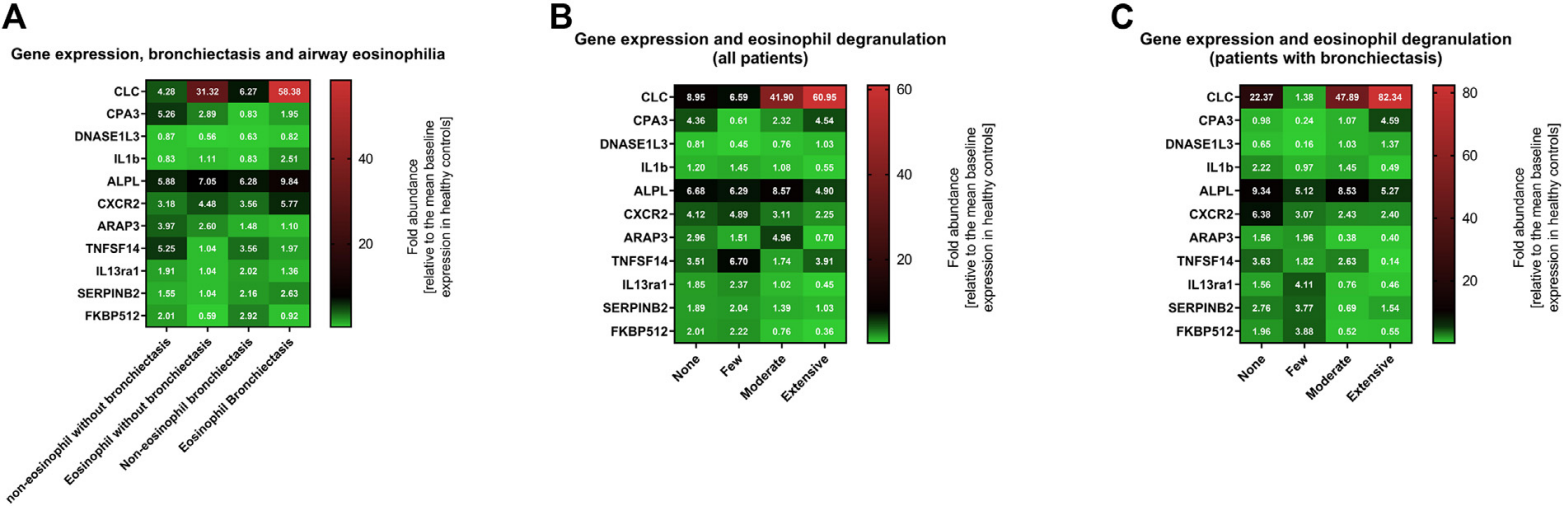
Heatmaps illustrating differences in gene expression based on (**A**) the presence of airway eosinophilia and bronchiectasis, (**B**) eosinophil degranulation across all patients, and (**C**) eosinophil degranulation in patients with bronchiectasis. Gene expression is presented as mean fold abundance in mean gene expression relative to the mean of a healthy control group. A fold abundance of more than 1 equals a relative increase compared with the mean in healthy control group, whereas fold abundance less than 1 equals a relative decrease.

Using multiple logistic regression, we found that no single gene was significantly associated with the presence of bronchiectasis; however, patients with bronchiectasis had a significantly higher expression of CLC (*P* = .024) compared with those without bronchiectasis (Fig 3, *A*), whereas there was no significant difference in expression of the other assessed genes. In exploratory analyses, we found differences in CLC (*P* = .01) and carboxypeptidase A3 (*P* = .03) expression when stratifying for eosinophil degranulation; however, these were not statistically significant when correcting for multiple comparisons (Fig 3, *B*). A similar pattern was observed for CLC in patients with bronchiectasis (Fig 3, *C*), but again this was not statistically significant after correcting for multiple comparisons.

## Discussion

We found bronchiectasis to be prevalent in one-third (31%) of patients with severe asthma and to be associated with eosinophil airway inflammation and activation, with extensive eosinophilic degranulation. Overall, our findings indicate that eosinophilic airway inflammation is an important cause of bronchiectasis in severe asthma, and support that this is related to an increased activation of airway eosinophils, with degranulation with release of cytotoxic enzymes such as EPX.

At present, our understanding of the inflammatory mechanisms resulting in eosinophil bronchiectasis (with and without severe asthma) is limited and no studies have so far used airway inflammometry to describe the inflammatory process(es). Contrary to the general paradigm of bronchiectasis as a neutrophilic phenomenon, we found that half the patients with bronchiectasis had eosinophilic airway inflammation and that 59% of patients with eosinophilic airway inflammation had bronchiectasis. In addition, we found no difference in the expression levels of the T_H_1/T_H_17-associated genes (IL-1β, ALPL, and C motif chemokine receptor 2) in patients with and without bronchiectasis.

These findings are in line with those of Coman et al^7^ who recently pointed out that patients with bronchiectasis and severe asthma had more frequent hospitalizations, hypersensitivity to nonsteroidal anti-inflammatory drugs, and a higher blood eosinophil count.

In line with the promising results of IL-5–targeted therapy for bronchiectasis without asthma reported by Rademacher et al,^26^ we found patients with bronchiectasis to have significantly elevated levels of sputum eosinophils, which have been linked with a predominantly IL-5–driven disease. In continuation, patients with bronchiectasis had a significantly higher, albeit only using single comparison and not multiple regression, level of messenger RNA expression of CLC—which has been linked to mucus plug formation—and been reported increased in the peripheral blood of patients with aspirin-induced asthma and in the nasal lavage fluid of patients with aspirin-sensitive respiratory disease, which widely are regarded as traits of the predominantly IL-5–related late-onset eosinophilic phenotype.^27^, ^28^, ^29^, ^30^, ^31^

Previous studies have suggested that IL-13–driven mucus hypersecretion could be a key mechanism in eosinophilic bronchiectasis.^8^ In line with previous findings, we found symptoms of chronic bronchitis to be significantly more prevalent in patients with BE; however, contrary to Dunican et al,^8^ we found no evidence pointing to an increased level of IL-13 activity in patients with bronchiectasis using conventional (fractional exhaled nitric oxide) and molecular inflammometry (messenger RNA expression).^8^ This finding is in line with that by Tsikrika et al,^6^ who reported an elevated IL-13 level in sputum in patients with BE but also noted that the IL-13 level was significantly lower compared with that in a reference population of patients with severe refractory asthma (n = 40).

Eosinophil-driven oxidation has recently been suggested as a key mediator of mucus plug formation in asthma.^8^ In particular, EPO—which is the most abundant granule protein—is considered key in the generation of reactive oxidants that can kill pathogens or activate airway cells, including mast cells.^32^

Recently, Dunican et al^8^ reported higher levels of EPO in patients with asthma with mucus plugs and linked EPO increased elasticity of airway mucus gels.

Supporting these findings, we report that eosinophil degranulation plays a role in bronchiectasis in patients with severe asthma, because “extensive” eosinophil degranulation was significantly more prevalent in patients with bronchiectasis but larger studies confirming this finding are warranted.

In continuation, we speculate that treatment with D_4_ leukotrien-receptor antagonists—which inhibit eosinophil degranulation^33^—may be beneficial in patients with bronchiectasis and severe asthma albeit this hypothesis is limited by the cross-sectional design of our study.

In summary, our cross-sectional molecular analyses support the notion of eosinophilic bronchiectasis as predominantly IL-5 driven; however, our statistical power is weak and our findings should be interpreted accordingly.

Future prospective studies must address whether targeting the IL-5 pathway alone is sufficient and we speculate that IL-13, through mucus hypersecretion, still may play a role in the development and progression of bronchiectasis, which ultimately may warrant blocking of both pathways, potentially through upstream alarmins such as TSLP or IL-33.^34^

As is to be expected given the definitions, we observed a marked difference in the magnitude of eosinophilic inflammation in patients with purely eosinophilic inflammation compared with those with mixed granulocytic inflammation. The importance of these differences is unknown, but they could reflect differences in the underlying mechanism, for example, alterations in the microbiome in the latter; however, we are unable to address this issue given our small sample size.

So far, the reported prevalence of bronchiectasis in severe asthma has varied markedly, which we believe largely can be attributed to selection bias. In contrast, our study was designed to reflect a clinically representative population of patients with severe asthma and all patients were assessed for bronchiectasis independent of clinical presentation, which we believe is a marked strength of our study.

At present, no standardized classification exists for the severity of bronchiectasis in asthma. We have in this article adapted the radiologic stratification from the Bronchiectasis Severity Index; however, we have chosen not to calculate a Bronchiectasis Severity Index score.^15^ We chose to do so, because we are unable to distinguish the cause of exacerbations and hospitalizations, which both contribute individually to the Bronchiectasis Severity Index score, and therefore believe that the established strong association between eosinophilia and frequent exacerbations would heavily bias analyses of the score in relation to our hypothesis. Furthermore, no data on microbiological colonization were available.

The aim of this article was to describe bronchiectasis in severe asthma, and we were cautious of the risk of misdiagnosing ABPA as severe asthma with concomitant bronchiectasis. Ten percent of our study population was sensitized to *A fumigatus*, with a significantly higher prevalence in patients with bronchiectasis; however, only 3% of patients had concomitant marked elevation of total IgE and *A fumigatus* sensitization. We do not believe our results to be confounded by undetected ABPA because all our significant associations remained significant when adjusting for sensitization for *A fumigatus*. Furthermore, no parameters were significantly different between patients with sensitization to *A fumigatus* and bronchiectasis compared with those with bronchiectasis alone albeit this analysis is severely limited by the low n.

Induced sputum is the criterion standard for airway inflammometry in asthma but does not, contrary to the more invasive bronchoschopic sampling, allow for targeted sampling of specific areas of the lung. Because bronchiectasis is often restricted to certain areas of the lung,^8^ we acknowledge a risk of bias due to spatio-inflammatory heterogeneity.^35^ To accommodate this risk of bias, we believe that future mechanistic studies should consider using computed tomography–guided bronchoscopic airway inflammometry preferably using the cryo-technique, which yields bigger and more intact bronchial biopsies with a higher content of deeper lying structures.^36^

Induced sputum was missing in 26% of patients; however, because only sputum at follow-up, and not at baseline, was found to be not-missing-at-random in a recent publication on the missingness of sputum, we do not consider this to have introduced any significant bias.^37^

In conclusion, bronchiectasis is a highly prevalent comorbidity in this generalizable population of patients with severe asthma, and our findings point to an increased activation of airway eosinophils in bronchiectasis, with extensive degranulation significantly more prevalent. We believe our findings highlight the glaring need for a better understanding of the inflammatory processes underlying bronchiectasis in severe asthma.

**Key messages**

• In this generalizable population of patients with severe asthma, bronchiectasis was a highly prevalent (31%) comorbidity predominantly associated with eosinophilic airway inflammation.
• Eosinophilic airway inflammation is an important cause of bronchiectasis in severe asthma, and our findings point to an increased activation of airway eosinophils with degranulation as a potential mechanism.
